# DNA repair gene XRCC1 Arg194Trp polymorphism and susceptibility to hepatocellular carcinoma: A meta-analysis

**DOI:** 10.3892/ol.2014.2351

**Published:** 2014-07-15

**Authors:** WENYAN LI, FENG YANG, YONGXIAN GUI, JUNJIE BIAN

**Affiliations:** 1 Department of Oncology, Xinxiang Central Hospital, Xinxiang Medical College, Xinxiang, Henan 453000, P.R. China; 2 Department of Integrated Medicine, Henan Cancer Hospital, Zhengzhou, Henan 450000, P.R. China

**Keywords:** X-ray repair cross-complementing group 1, gene polymorphism, hepatocellular carcinoma

## Abstract

The arginine194tryptophan (Arg194Trp) polymorphism in the X-ray repair cross-complementing group 1 (XRCC1) gene has been reported to be associated with hepatocellular carcinoma (HCC), however, the results from previous studies are conflicting. The present study aimed to investigate the association between the XRCC1 Arg194Trp polymorphism and the risk of HCC, using a meta-analysis of previously published studies. PubMed (http://www.ncbi.nlm.nih.gov/pubmed/), Google Scholar (http://scholar.google.co.uk/) and the China National Knowledge Infrastructure databases (http://www.cnki.net/) were systematically searched to identify relevant studies published prior to October 2013. A meta-analysis was performed to examine the association between the Arg194Trp gene polymorphism and the susceptibility to HCC. Odds ratios (ORs) and 95% confidence intervals (95% CIs) were calculated. The meta-analysis consisted of six case-control studies that included 1,451 HCC cases and 1,398 healthy controls. Meta-analysis results based on all the studies showed no significant association between the XRCC1 Arg194Trp gene polymorphism and the risk of HCC (Trp/Trp vs. Arg/Arg: OR, 1.17; 95% CI, 0.89–1.55; Trp/Trp vs. Arg/Trp: OR, 0.94; 95% CI, 0.59–1.51; dominant model: OR, 0.97; 95% CI, 0.63–1.49; recessive model: OR, 1.22; 95% CI, 0.89–1.67). In the subgroup analysis, three studies with sample sizes of >300 produced similar results that indicated that the Arg194Trp gene polymorphism had no association with an increased or decreased risk of HCC. The pooled ORs were not markedly different following the exclusion of two studies deviating from the Hardy-Weinberg equilibrium in the control group, which indicated the reliability of the meta-analysis results. In conclusion, the XRCC1 Arg194Trp polymorphism may not be a risk or protective factor for HCC. Further large and well-designed studies are required to confirm these results.

## Introduction

Hepatocellular carcinoma (HCC) is a common primary liver cancer with a rising incidence globally (1). The estimated number of new cases of HCC is ~564,000 per year worldwide (2). Human HCC development and progression is a long-term, multi-step process that is correlated with the sequential evolution of stages that are morphologically distinct and result in fully developed HCC. The main risk factors for HCC are alcoholism, hepatitis B and C, aflatoxin, cirrhosis of the liver, hemochromatosis, Wilson’s disease and type 2 diabetes (3). Numerous studies have investigated the genes underlying the development and progression of HCC, and have proposed that the pathogenesis of HCC may be affected by multiple genetic factors (4–6).

Located on chromosome 19 (19q13.2), the X-ray repair cross-complementing group 1 (XRCC1) gene is known to encode a vital scaffold protein that has close associations with the base excision repair (BER) pathway (7). The functions of the BER pathway in the process of DNA repair require the use of the XRCC1 protein, which has a significant role in genome integrity and stability, and in human cancer pathogenesis and progression (8). Although >300 validated single nucleotide polymorphisms (SNPs) have been identified and described in the XRCC1 gene, only three common SNPs have been extensively studied: Argenine399glutamine (Arg399Gln; rs25487, G/A substitution at position 28,152 on exon 10), argenine280histidine (Arg280His; rs25489, G/A substitution at position 27,466 on exon 9) and arginine194tryptophan (Arg194Trp; rs1799782, C/T substitution at position 26,304 on exon 6). These variations were shown to be able to alter the function of XRCC1, diminish repair kinetics and lead to altered protein efficiency, eventually inducing the development of cancer (9).

Previous studies have demonstrated that the XRCC1 Arg194Trp gene polymorphism is associated with the susceptibility to esophageal, gastric, lung, breast and other types of cancer (10–12). There is little known regarding the association between the XRCC1 Arg194Trp gene polymorphism and the susceptibility to HCC. Over the past decade, several case-control studies have focused on the association between the Arg194Trp gene polymorphism and the HCC risk, however, the results remain controversial (13–18). In the present study, a meta-analysis was performed to investigate whether the Arg194Trp gene polymorphism was associated with the risk of HCC.

## Materials and methods

### Identification and eligibility of relevant studies

Two researchers independently investigated the titles, abstracts and full texts of relevant studies to determine whether they could be included in the present study. The results were compared and disagreements were resolved by consensus. All case-control studies of the XRCC1 Arg194Trp gene polymorphism and HCC risk published up to October 25, 2013 were identified through systematic searches in PubMed (http://www.ncbi.nlm.nih.gov/pubmed/), Google Scholar (http://scholar.google.co.uk/) and the China National Knowledge Infrastructure (CNKI) databases (http://www.cnki.net/), using English and Chinese. The search terms used were: X-ray repair cross-complementing group 1, XRCC1, polymorphism, variation and mutation, plus all of these terms in combination with hepatocellular carcinoma, HCC, liver cancer, liver tumor, liver neoplasms and hepatic tumor. The reference lists of the retrieved articles were hand-searched to obtain other relevant publications. All associated publications were evaluated to identify the most eligible literature. Studies that were reported by the same authors were checked for possible overlapping participant groups.

### Inclusion and exclusion criteria

The inclusion criteria were as follows; i) Case-controlled studies that addressed HCC cases and healthy controls; ii) studies that evaluated the association between the XRCC1 Arg194Trp gene polymorphism and the HCC risk; iii) all patients with clinically diagnosed HCC; iv) studies that included sufficient genotype data for extraction; v) the studies contained at least two comparison groups (cancer group vs. control group); and vi) the studies included detailed genotyping data. The exclusion criteria were as follows: i) Not case-control studies that evaluated the association between the XRCC1 Arg194Trp gene polymorphism and the HCC risk; ii) animal studies; iii) studies that were based on incomplete raw data or no usable data reported; and iv) duplicated publications.

### Data extraction

Two investigators independently performed the extraction of data from all the eligible publications, according to the inclusion and exclusion criteria. Any discrepancy between the two investigators was settled by discussion until a consensus was reached. For each study, the following data were considered: First author’s name, year of publication, country of the study, ethnicity, numbers of genotyped cases and controls, and deviation from the Hardy-Weinberg Equilibrium (HWE) of the control group.

### Statistical methods

The HWE in the controls was assessed for each study using the χ^2^ test, and P<0.05 was considered to indicate a statistically significant disequilibrium. In the overall and subgroup meta-analyses, the strength of the association between the XRCC1 Arg194Trp gene polymorphism and the HCC risk was measured by odds ratios (ORs) and 95% confidence intervals (CIs). The combined ORs and 95% CIs were calculated respectively for a homozygote comparison (Trp/Trp vs. Arg/Arg), a heterozygote comparison (Trp/Trp vs. Arg/Trp), a dominant model (Arg/Arg + Arg/Trp vs. Trp/Trp) and a recessive model (Trp/Trp + Arg/Trp vs. Arg/Arg) between groups. The effect of heterogeneity was quantified using an I^2^ test. I^2^ ranged between 0 and 100% and represented the proportion of inter-study variability that could be attributed to heterogeneity rather than chance. I^2^ values of 25, 50 and 75% were defined as low, moderate and high estimates, respectively. When I^2^>50% indicated heterogeneity across studies, a random-effects model was used for the meta-analysis, otherwise a fixed-effects model was used (19,20). Publication bias was examined by plotting a Begg’s funnel plot, and P<0.05 was considered to indicate a statistically significant publication bias. Stratified analyses were performed by sample size (subjects >300) and P<0.05 was considered to indicate a statistically significant difference. To assess the reliability of the outcomes in the meta-analysis, a sensitivity analysis was performed, excluding studies whose allele frequencies in the controls exhibited a significant deviation from the HWE. All statistical tests were performed using STATA v.12.0 software (Stata Corporation, College Station, TX, USA).

## Results

### Study selection

**Figure 1:**
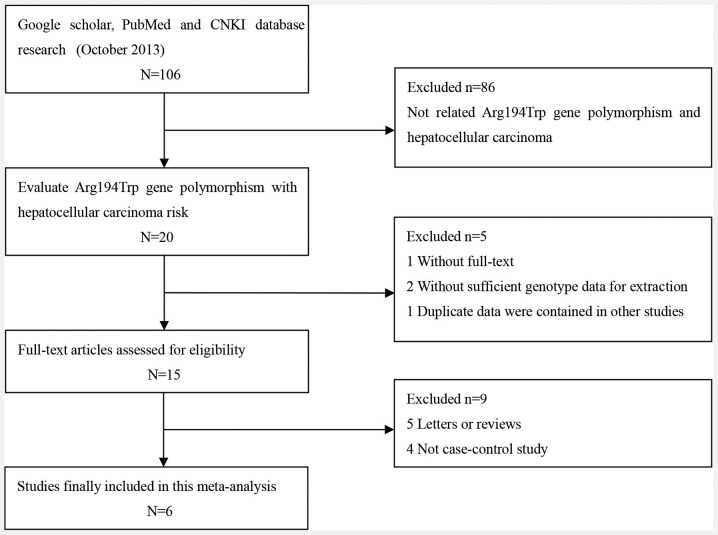
Flow chart of the study selection based on the inclusion and exclusion criteria. Arg, arginine; Trp, tryptophan.

A total of 106 potentially relevant publications were systematically identified through a search of PubMed, Google Scholar and CNKI up to October 2013. Based on the preliminary search criteria, 100 studies were excluded as they did not satisfy the inclusion criteria. In total, 1,451 cases and 1,398 controls were included in the meta-analysis. The study characteristics are summarized in Fig. 1 and Table I. The six studies were all of individuals of Asian descent, from which, five were from China and one was from India (13–18). The distribution of the genotypes in the controls was consistent with the HWE in all studies except for that of Bo *et al* (16) and Guo *et al* (17). Furthermore, three studies that were conducted with >300 subjects were included in the subgroup meta-analysis (14,17,18). Of these six studies, one used TaqMan probe methodology and five used a polymerase chain reaction-restriction fragment length polymorphism method to identify the XRCC1 SNPs.

### Quantitative data synthesis

**Figure 2:**
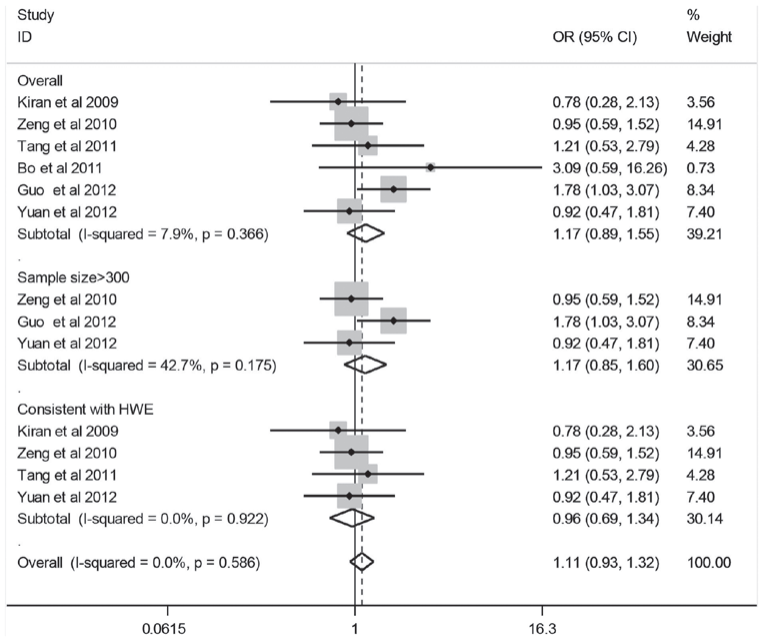
Forest plots of the Arg194Trp gene polymorphism in the hepatocellular carcinoma vs. normal control and subgroup analyses. HWE, Hardy-Weinberg equilibrium; OR, odds ratio; CI, confidence interval; Arg, arginine; Trp, tryptophan.

The results of the associations between the Arg194Trp gene polymorphism and the HCC risk, the heterogeneity test and the test of publication bias are shown in Fig. 2 and Table II. The combined results based on all the studies showed that variant genotypes were not associated with an increased HCC risk in different genetic models (Trp/Trp vs. Arg/Arg: OR, 1.17; 95% CI, 0.89–1.55; Trp/Trp vs. Arg/Trp: OR, 0.94; 95% CI, 0.59–1.51; dominant model: OR, 0.97; 95% CI, 0.63–1.49; recessive model: OR, 1.22; 95% CI, 0.89–1.67). In the stratified analysis by sample size (subjects, >300), no significant association was identified between the Arg194Trp gene polymorphism and HCC (Trp/Trp vs. Arg/Arg: OR, 1.17; 95% CI, 0.85–1.60; Trp/Trp vs. Arg/Trp: OR, 1.07; 95% CI, 0.78–1.49; dominant model: OR, 0.88; 95% CI, 0.65–1.19; recessive model: OR, 1.11; 95% CI, 0.86–1.44).

### Tests of heterogeneity

Statistically significant heterogeneity was observed between the trials of the following analyses using the I^2^ test (Trp/Trp vs. Arg/Trp: I^2^=59.2%, P=0.03; dominant model: I^2^=57.0%, P=0.04; recessive model: I^2^=72.3%, P=0.00) (Table II), and a random-effects model was employed in these studies. There was no significant heterogeneity identified for Trp/Trp vs. Arg/Arg (I^2^=7.9%, P=0.37) after performing a fixed-effects model.

### Sensitivity analysis

A sensitivity analysis was performed following the removal of the studies by Bo *et al* (16) and Guo *et al* (17) due to the genotype distribution in the control groups deviating from the HWE. The results suggested that no individual study significantly affected the pooled ORs, although in certain cases, the I^2^ value for heterogeneity was reduced. The sensitivity analysis therefore confirmed that the data of this meta-analysis was statistically robust.

### Publication bias

The funnel plot and Begg’s test was used to assess the publication bias of the selected literature. No evidence of publication bias was detected in the study, and therefore publication bias was low in the present meta-analysis (all P>0.05). Information concerning the Begg’s funnel plot is shown in Table II.

## Discussion

The estimated incidence of new HCC cases each year is >0.5 million (19), with a wide geographic variation in incidence regions at an international level; a high incidence can be found in Eastern and South-Eastern Asia, while a low incidence can be observed in developed regions (20). It is well-known that hepatitis B virus is the predominant risk factor for the pathogenesis of HCC. In addition, epidemiological investigations have demonstrated that the occurrence and development of HCC has a strong genetic predisposition (23). The XRCC1 protein is vital in the multistep nucleotide excision repair pathway, and it is the first mammalian gene to be isolated that affects the sensitivity of cells to ionizing radiation (24). Various studies have focused on the association between the Arg194Trp gene polymorphism and HCC. However, the observed associations of these studies were inconclusive (13–18). The reason for the inconsistencies among these studies is most likely to be that they were single, small-sample, case-control studies. To help resolve these conflicting results, the present study performed a meta-analysis to combine the study types in order to increase the sample size and statistical power.

A meta-analysis technique was used to collect comparable published or unpublished data, and statistical methods were applied to synthesize the independent results of the studies with the same research target, in order to obtain a combined quantitative conclusion. This method could provide scientific, repeatable and objective reasoning as to why similar studies produced different results (25,26). The present meta-analysis, including 1,451 cases and 1,398 controls from six case-control studies, explored the association between the Arg194Trp XRCC1 gene polymorphism and the HCC risk.

The results of the present meta-analysis revealed that the XRCC1 Arg194Trp gene polymorphism was not associated with an increased or decreased risk of HCC. However, a previous study by Kiran *et al* (13), reported that this same polymorphism increased the risk of susceptibility to HCC in Indian patients with hepatitis (OR, 2.27; 95% CI, 1.01–5.08). This difference in result may be associated with ethnic and regional differences. The present meta-analysis also involved several studies with a small sample size; there may have been a selective bias for the association between the XRCC1 Arg194Trp gene polymorphism and HCC development, and therefore, large-sample studies should be used to re-evaluate this association. When stratifying by sample size (>300), the present meta-analysis detected no significant association, indicating that there was no evidence of a small-study bias in the meta-analysis. Further sensitivity analysis confirmed the significant association between the maternal XRCC1 Arg194Trp gene polymorphism and the HCC risk. There was no evidence to suggest a publication bias in the present meta-analysis for the XRCC1 Arg194Trp gene polymorphism (P>0.05).

The effect of the XRCC1 Arg194Trp gene polymorphism may have a limited impact on HCC. As with other malignant tumors, the development of HCC is due to the combined effect of multiple genes and gene-environment interactions (27). Previous data have suggested that for the combination of XRCC1 Arg194Trp and Arg280His or Arg399Gln, there is a markedly increased risk of hepatitis-related HCC (13). Furthermore, the risk of HCC for the XRCC1 Arg194Trp genotype is 1.29 times higher than that of the XRCC1 194Arg genotype with exposure to alcohol. Drinking may therefore increase the HCC risk, although there appears to be no significant difference between the genotypes (P>0.05) (18). Further studies of gene-gene and gene-environment interactions should be taken into consideration in future analyses, which should lead to an improved, comprehensive understanding of the association between the XRCC1 Arg194Trp gene polymorphism and the HCC risk.

Certain limitations of the present meta-analysis should be acknowledged in order to establish a complete interpretation of the data. Firstly, the present meta-analysis was based on unadjusted OR estimates since not all the published studies presented adjusted ORs. In cases where the adjusted OR was presented, they were not adjusted by the same potential confounders, such as age, gender, ethnicity and exposures. A lack of information for the data analysis may cause a confounding bias. Secondly, the number of studies and the number of subjects in the studies included in the meta-analysis by specific subgroups were small. Thirdly, a lack of original data limited a further evaluation of the potential gene-gene and gene-environment interactions.

In conclusion, the present meta-analysis suggested that the XRCC1 Arg194Trp gene polymorphism may be not associated with the HCC risk. Further studies estimating the effects of gene-gene and gene-environment interactions may provide an improved comprehensive understanding of the association between XRCC1 and the HCC risk.

## Figures and Tables

**Table I:** Characteristics of literature studies included in the meta-analysis.

					Genotypes for cases			Genotypes for controls			
							
First author (ref.)	Year	Area	Race	Cases/controls	Arg/Arg	Arg/Trp	Trp/Trp	Arg/Arg	Arg/Trp	Trp/Trp	HWE test
Kiran *et al* (14)	2009	India	Asian	63/143	8	43	12	27	64	52	0.35
Zeng *et al* (15)	2010	China	Asian	545/515	305	200	40	275	202	38	0.91
Tang *et al* (16)	2011	China	Asian	147/150	91	41	15	81	58	11	0.89
Bo *et al* (17)	2011	China	Asian	130/130	94	31	5	116	12	2	0.02
Guo *et al* (18)	2012	China	Asian	314/210	264	109	37	292	96	23	0.00
Yuan *et al* (19)	2012	China	Asian	252/250	119	115	18	128	101	21	0.86

Arg, arginine; Trp, tryptophan; HWE, Hardy-Weinberg equilibrium.

**Table II:** Summary of ORs and 95% CIs of the Arg194Trp gene polymorphism and HCC risk.

		Sample size, n			Test of heterogeneity		Test of association		Test of publication bias	
						
Subgroup	Genetic model	Case	Control	Type of model	I^2^ (%)	P-value	OR	95% CI	z	P-value
Overall	Trp/Trp vs. Arg/Arg	1451	1398	Fixed	7.9	0.37	1.17	0.89–1.55	0.00	1.00
	Trp/Trp vs. Arg/Trp			Random	59.2	0.03	0.94	0.59–1.51	0.00	1.00
	Dominant model			Random	57.0	0.04	0.97	0.63–1.49	0.00	1.00
	Recessive model			Random	72.3	0.00	1.22	0.89–1.67	0.00	1.00
Sample size >300	Trp/Trp vs. Arg/Arg	1111	975	Fixed	42.7	0.18	1.17	0.85–1.60	1.04	0.30
	Trp/Trp vs. Arg/Trp			Fixed	0.0	0.40	1.07	0.78–1.49	1.04	0.30
	Dominant model			Fixed	36.0	0.21	0.88	0.65–1.19	1.04	0.30
	Recessive model			Random	57.1	0.10	1.11	0.86–1.44	1.04	0.30
Consistent with HWE	Trp/Trp vs. Arg/Arg	1007	1058	Fixed	0.0	0.92	0.96	0.69–1.34	0.34	1.00
	Trp/Trp vs. Arg/Trp			Random	70.4	0.02	0.84	0.45–1.57	0.34	1.00
	Dominant model			Fixed	49.9	0.11	1.19	0.88–1.60	0.34	1.00
	Recessive model			Fixed	31.0	0.23	0.36	0.80–3.15	0.34	1.00

Arg, arginine; Trp, tryptophan; HWE, Hardy-Weinberg equilibrium; CI, confidence interval; OR, odds ratio; HCC, hepatocellular carcinoma.
